# Models of integrated care for young people experiencing medical emergencies related to mental illness: a realist systematic review

**DOI:** 10.1007/s00787-022-02085-5

**Published:** 2022-09-24

**Authors:** Michaela Otis, Susan Barber, Mona Amet, Dasha Nicholls

**Affiliations:** 1 Imperial College London, South Kensington, London, SW7 2BX UK; 2 NIHR Applied Research Collaboration (ARC) Northwest London, London, UK; 3 Chelsea and Westminster NHS Foundation Trust, London, UK

**Keywords:** Emergency medicine, Paediatrics, Integrated care, Psychological intervention, Healthcare utilization, Psychiatry

## Abstract

Mental illness heightens risk of medical emergencies, emergency hospitalisation, and readmissions. Innovations for integrated medical–psychiatric care within paediatric emergency settings may help adolescents with acute mental disorders to get well quicker and stay well enough to remain out of hospital. We assessed models of integrated acute care for adolescents experiencing medical emergencies related to mental illness (MHR). We conducted a systematic review by searching MEDLINE, PsychINFO, Embase, and Web of Science for quantitative studies within paediatric emergency medicine, internationally. We included populations aged 8–25 years. Our outcomes were length of hospital stay (LOS), emergency hospital admissions, and rehospitalisation. Limits were imposed on dates: 1990 to June 2021. We present a narrative synthesis. This study is registered on PROSPERO: 254,359. 1667 studies were screened, 22 met eligibility, comprising 39,346 patients. Emergency triage innovations reduced admissions between 4 and 16%, including multidisciplinary staffing and training for psychiatric assessment (F(3,42) = 4.6, *P* < 0.05, *N* = 682), and telepsychiatry consultations (aOR = 0.41, 95% CI 0.28–0.58; *P* < 0.001, *N* = 597). Psychological therapies delivered in emergency departments reduced admissions 8–40%, including psychoeducation (aOR = 0.35, 95% CI 0.17–0.71, *P* < 0.01, *N* = 212), risk-reduction counselling for suicide prevention (OR = 2.78, 95% CI 0.55–14.10, *N* = 348), and telephone follow-up (OR = 0.45, 95% CI 0.33–0.60, *P* < 0.001, *N* = 980). Innovations on acute wards reduced readmissions, including guided meal supervision for eating disorders (*P* = 0.27), therapeutic skills for anxiety disorders, and a dedicated psychiatric crisis unit (22.2 vs 8.5% (*P* = 0.008). Integrated pathway innovations reduced readmissions between 8 and 37% including family-based therapy (FBT) for eating disorders (*X*^2^(1,326) = 8.40, *P* = 0.004, *N* = 326), and risk-targeted telephone follow-up or outpatients for all mental disorders (29.5 vs. 5%, *P* = 0.03, *N* = 1316). Studies occurred in the USA, Canada, or Australia. Integrated care pathways to psychiatric consultations, psychological therapies, and multidisciplinary follow-up within emergency paediatric services prevented lengthy and repeat hospitalisation for MHR emergencies. Only six of 22 studies adjusted for illness severity and clinical history between before- and after-intervention cohorts and only one reported socio-demographic intervention effects.

**Supplementary Information:**

The online version contains supplementary material available at 10.1007/s00787-022-02085-5.

## Introduction

Mental disorders are experienced by 10% of children and adolescents globally [1], and 16% in the UK [2]. Physical health emergencies often have mental health-related (MHR) aetiologies, exacerbators, and consequences, indicating that mental and physical illnesses are interrelated [3]. Comorbid mental disorders are present in 10–19% of all paediatric emergencies [4, 5], and mental illness significantly increases risk of medical emergency admission and readmissions [6]. Frequent attenders at paediatric emergency departments more commonly present with psychiatric complaints than with accidental injury [7]. Examples of physical manifestations of mental disorders are self-harm and suicide attempts, psychological trauma leading to somatisation, eating disorders leading to serious nutritional deficiencies, and substance and alcohol dependence leading to intoxication [5]. This exemplifies how some MHR emergencies are inherently multimorbid and require integrated paediatric and psychiatric care. Fifty percent of all lifetime mental disorders have an onset prior to 14 years of age [8], and mental illness hinders social, educational, and occupational development. [9] Thus, there is a need to intervene effectively at the critical moment when adolescents present with MHR emergencies, to prevent persistent and pervasive complications [10].

MHR emergencies require a multidisciplinary response to comprehensively meet the biopsychosocial needs of young people. National and local health policies in the UK advocate for integrated care, through collaborations between providers, intervening early, and evidence-based mental health services [11–13]. At a regional level, introducing multidisciplinary teams and cross-service planning reduced the average length of inpatient stay 22% and admissions 7% [14]. Whilst local targets for integrated emergency care for children and adolescents were established in 2019, challenges in collaborative commissioning persist. This informed our research objective to evidence effective models of care to encourage investment.

Mental illness is often lifelong [15], and 8–13% of those hospitalised in paediatric settings return within one month, significantly higher than non-MHR admissions [4, 16]. The effectiveness of therapeutic modalities on improving health outcomes is well documented. [17] For example, integrating family-based therapy within acute hospitalisation has been provided in medical emergencies that are accompanied by a comorbid mental illness—particularly eating disorders and suicide attempts. This is known as partial hospitalisation, which involves transferring the patient from an inpatient ward to outpatient family-based therapy once medical stabilisation is complete; aimed at reducing the degree of institutionalisation [18]. Integrated family-based therapy as partial hospitalisation improved young people’s psychosocial functioning and parental self-efficacy to support the young person to stay well. This review adopted the hypothesis that providing family-based therapy as an integrated intervention from acute hospitalisation for MHR emergencies would reduce repeat hospitalisation, helping young people to stay out of hospital. Other types of integrated psychological interventions are aimed at supporting young people to stay out of hospital after an MHR emergency, such as outpatient follow-up with a psychiatrist, or individual therapy, which were also included in this review. We know that these interventions are health effective, but we do not yet know whether they are effective for health maintenance. Preferably, evaluations will test for sustained health outcomes, but patient data is often not recorded centrally after leaving the hospital. This highlights the need to monitor admissions, readmissions, and length of hospital stay (LOS), as proxies for efficient and sustained recovery, with minimal institutionalisation. Recent evidence indicates that integrating multidisciplinary staffing to provide both medical and psychiatric triage, as well as delivering low-intensity psychological therapy within acute psychiatric settings improved service efficiency and treatment capacity [19]. Yet, there is limited evidence whether these therapies improve healthcare efficiency when integrated into paediatric emergency settings.

Innovations for integrated care implies that the individual received both medical and psychological emergency care in one care-package, i.e. initiated within one emergency care visit. This optimises patients’ time rather than visiting multiple providers for different aspects of care. When facilities do not allow integrated care provision in one location, it may be necessary to supplement care with additional specialist services. Thus, the term ‘innovation’ refers to establishing models of integrated care that are delivered by, or integrated alongside, emergency departments and acute hospitalisation. One example of integrated care is providing psychological assessment and treatment, subject to the patient being well enough to participate, alongside acute medical care [20]—instead of prolonged hours of unstructured time and social isolation on an acute ward. These innovations have implications not only for improving health outcomes [11, 17], but most importantly to avoid institutionalisation by minimising time spent in hospital and supporting return to a home environment [17].

We identified an evidence gap—whether embedding psychiatry within paediatric emergency settings improves health maintenance to stay out of hospital. We present an evidence synthesis and implications for clinical practice and research.

## Methods

### Search strategy and selection criteria

This systematic review adhered to PRISMA guidelines [21]. We included quantitative observational studies published in peer-reviewed journals that evaluated innovations located within acute paediatric hospital services, such as the emergency department, emergency admission wards, emergency crisis centres, intensive care wards, and emergency referrals from these acute services to outpatient services. We included both innovations for integrated care provision and integrated care pathways, whenever the care was delivered as emergency healthcare. This included any intervention that provided psychological care provided by mental health practitioners, such as family-based therapy, psychiatric follow-up or aftercare, or individual therapy, either within, or integrated alongside, emergency healthcare. Given that intensive therapeutic interventions delivered alongside acute hospitalisation are conceptually different from brief psychological support delivered in emergency departments and inpatient wards, we also extracted the staffing requirements and intervention duration for all studies reviewed—important considerations for cost–benefit analysis. We included study samples up to 25 years of age [22] if the study also included those aged below 18 years. The outcomes of interest were emergency admissions, emergency rehospitalisation, and LOS in acute settings. All study designs using a control group were included.

We defined participants as presenting with MHR emergencies, which are identified symptomatically and behaviourally, considering that underlying mental illnesses are not necessarily diagnosed. Hence, we included cohorts of either MHR emergency presentations (i.e, self-harm, suicide attempts, and intoxication) as well as specific mental illnesses (i.e. eating disorders, substance abuse and alcohol-related disorders, mood disorders, neurodevelopmental disorders, and psychotic disorders). These groups were informed by the ICD-11 and a service evaluation for MHR paediatric emergencies [5].

The search strategy was developed in correspondence with a university librarian. We searched MEDLINE (May 28, 2021), PsycINFO (Jun 10, 2021), Embase (Jun 10, 2021), and Web of Science (Jun 15, 2021) for published articles. We searched database subject headings and free-text keywords using a four-concept strategy consisting of (i) young people aged < 26 years, (ii) acute hospital settings (i.e. acute paediatrics, emergency medicine, and emergency department), (iii) with symptoms indicating mental illness, and (iv) admissions, LOS, or readmissions. We iteratively updated the search as new terms were identified in key texts found from the search. We imposed a date restriction from the year 1990 to current. Search strategies are provided in supplementary file 1.

Search results were de-duplicated in Zotero and again in Covidence software for systematic reviews. Two reviewers (MO and MA) identified studies that met inclusion criteria through screening of all titles and abstracts, then full-text screening using Covidence. Any discrepancies were resolved through consensus after discussion between both reviewers.

### Data extraction and quality assessment

A Microsoft Excel sheet was used to extract study designs, country, dates, sample sizes, participant characteristics (including gender, age, ethnicity, and diagnoses), innovation and comparator characteristics, and outcome measures (numeric and risk ratios). Two reviewers (MO and MA) blindly assessed the quality of extracted studies using an adapted version of the Newcastle–Ottawa Scale (NOS) for observational designs [23]. The NOS item measuring absence of outcome at the start of the study was removed given it was not relevant to our extracted studies, which all used retrospective data. We added two questions to the ‘comparability’ section of the NOS, adopted from questions 10 and 11 of the National Heart, Lung and Blood institute’s (NHLBI) ‘Quality assessment tool for before–after studies with no control group’ [24], assessing the appropriate time frame of outcome measurement to capture intervention effects and statistical comparisons. Quality assessment scores had an 85% interrater reliability. Discrepancies were resolved through discussion to agree to a final score. Quality assessment scores are provided in supplementary table 2.

All 22 studies used retrospective designs with routinely collected hospital data, comprising 16 before–after intervention designs, three non-randomised control trials, and three retrospective observational designs. Thus, all samples were complete in representing the population of young people using those services. Six studies were rated as good quality [25–30], 12 were fair quality [31–42], and 4 were poor quality [43–46]. There was considerable variability between study methodologies, particularly the post-intervention time frame to capture the intervention effects. The main area of weakness was the comparability of intervention and control groups, whereby four studies provided no statistical calculation, only six controlled for potential confounders, and only three used a rigorous method such as propensity score matching or interrupted time series analysis [25, 27, 38]. All studies were included in the narrative synthesis.

### Data analysis

We grouped studies by emergency care setting and innovation type to investigate the effect of healthcare innovations in each setting. A narrative synthesis was conducted for (i) triage in the emergency department, (ii) psychological therapy in the emergency department, and (iii) psychological therapy on the inpatient ward or provided as an integrated pathway referral. The narrative synthesis followed guidance for systematic reviews [47] to critique the evidence for effectiveness of interventions—i.e. what interventions (mechanisms) were effective (outcomes) for whom, in what settings and locations (contexts). This also identified evidence gaps. Due to heterogeneity in intervention types in the final set of studies, a meta-analysis was not considered.

## Results

**Fig. 1:**
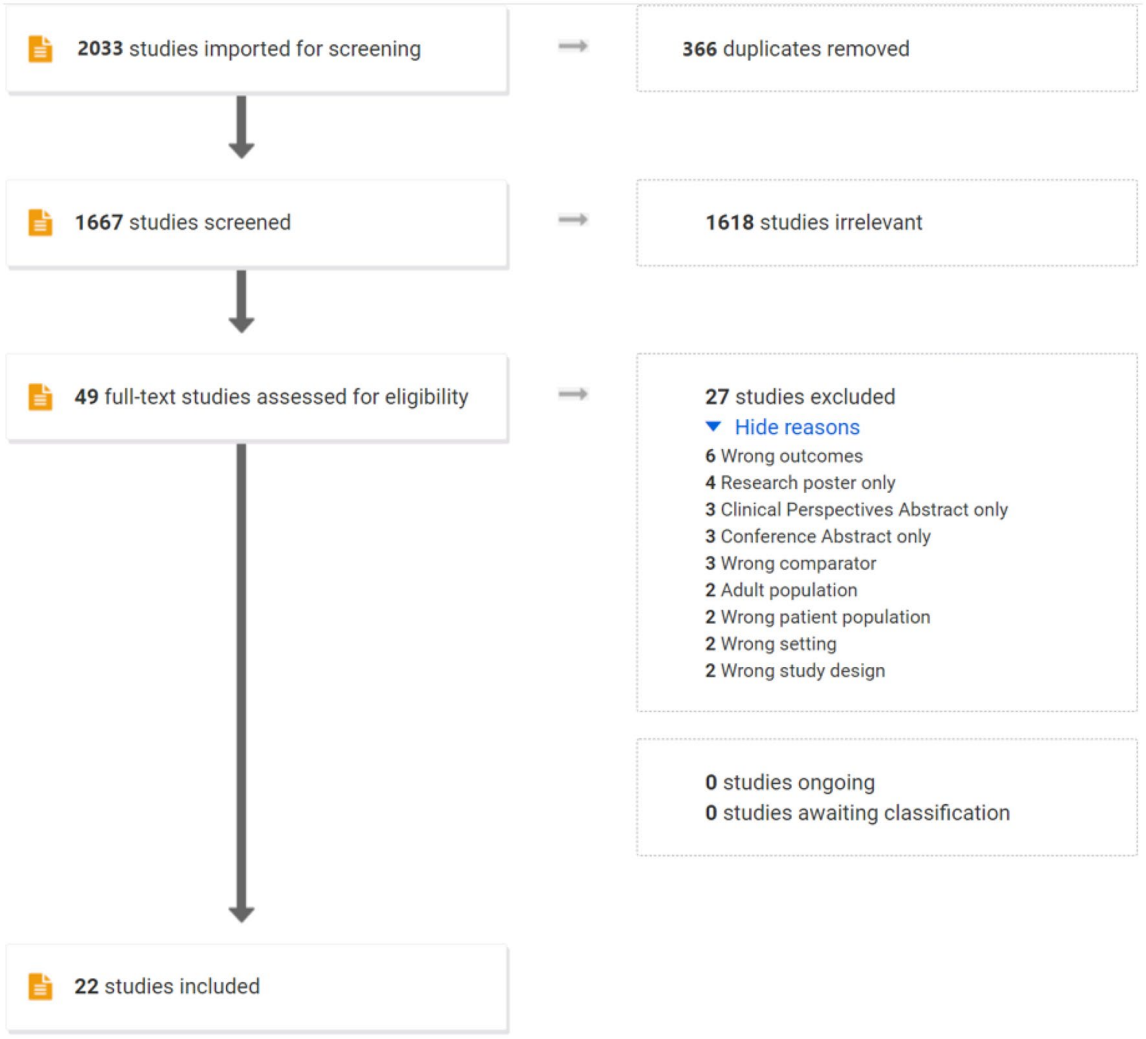
PRISMA flowchart of study selection

Our initial search generated 2033 results, of which 366 were duplicates, resulting in 1667 studies to be screened. After title and abstract screening, 50 relevant articles were identified, of which 22 met inclusion criteria after full-text screening. No further studies were identified through forward and backward searches in the references. Figure 1 shows the PRISMA flowchart of study selection.

All studies were from high-income countries, with 15 in the United States of America (USA), six in Canada, and one in Australia. Six studies included participants over the age of 18 years. In total, 39,346 young people were included across all studies, of whom between 6 and 37% were admitted for emergency care, spending between 183 and 363 min in the emergency department. All participants were referred by the emergency department for assessment by a mental health practitioner. Measuring readmission after discharge ranged between 30 days and one year across all studies, resulting in a wide-ranging 16–71% of participants with emergency readmissions. All studies extracted clinical characteristics from patient records, recorded as ICD-9 or -10 codes [48], referral for psychiatric assessment or service, or surveyed caregivers on the presence of intentional self-injury. Most studies reported patient demographics, but only one stratified intervention effects by age. For outcomes, ten studies reported on emergency admissions, six reported LOS in the emergency department, six on LOS in inpatient setting, and six on inpatient readmissions after discharge. Factors significantly associated with emergency admissions in multivariate analyses were (i) at the service level, higher volume of emergency visits (incident rate ratio, IRR = 1.004, 95% CI 1.003–1.005), and proportion of MHR emergencies (IRR = 1.004, 95% CI 1.002–1.006) [27]; (ii) at the individual level, older age (OR = 1.17, 95% CI 1.03–1.33), suicidal ideation with or without self-harm (OR = 3.07 or 4.70, 95% CI 1.12–11.01), and clinical diagnoses (when compared to depression), including bipolar or other mood disorders (OR = 13.05, 95% CI 3.57–47.71), and oppositional defiant disorder or conduct disorder (OR = 3.88, 95% CI 1.11–13.58) [30].

The first set of innovations improved psychiatric triage in the emergency department, including telepsychiatry consultations (*N* = 2), multidisciplinary staffing (*N* = 3), and clinical guidance training (*N* = 1), which were effective in reducing LOS [27, 34, 37, 39] and emergency admissions (see Table 1) [26, 34, 38]. Innovations for staff restructuring formed a multidisciplinary team comprising child psychiatrists and mental health social workers, supplemented with staff training in psychiatric triage, which reduced admissions 4–16% and LOS by 85–150 min (*P* < 0.05). [27, 34] Multidisciplinary staffing for medical and psychiatric assessments supported a new referral pathway to an urgent care team, which reduced admissions from the emergency department from 6.3 to 2.3% (F(3,42) = 4.6, *P* < 0.05) [38]. Next, the use of telepsychiatry consultations in the emergency department reduced emergency admissions (OR = 0.42, 95% CI = 0.30–0.59, *P* < 0.01); which was sustained after accounting for small differences in age and diagnoses between pre- and post-intervention cohorts (adjusted OR = 0.41, 95% CI 0.28–0.58, *P* < 0.01) [26]. Yet, whilst admissions reduced, there was longer LOS in the emergency department by 1 h (183 vs 125 min, *P* < 0.01), arguably, a fair trade-off to avoid admission. However, this longer LOS may have been confounded by differences in staff and patient characteristics during the day shift (the intervention group) and the night shift (the control group who received face-to-face consultations), which were not accounted for. A counterargument is provided by another telepsychiatry consultation study, which used a rigorous study design of 3 months before–after intervention, reporting a reduction in LOS from 285 to 193 min *(P* = 0.03) [39]. Alternatively, introducing clinical guidance training without staff restructuring or telepsychiatry reduced LOS from 259 to 216 min (*P* = 0.01) [37], though admission rates were not studied.

**Table 1:** Characteristics of triage innovations within emergency departments

Study (Country)	Population/inclusion criteria	Study design	Dates	Intervention and resources	Participants	Comparator	Outcomes	Intervention effects
Desai [26] (USA)	Children and adolescents with a psychiatric disorder, aged 5–21 years	Non-randomised controlled trial	2016	Telepsychiatry consultations (between 8am and 4 pm daily, delivered by a child psychiatrist)	Total *N* = 597 (intervention *N* = 257, routine *N* = 340)	Face-to-face assessment (between 4 pm and 8am daily)	LOS in emergency dept. and emergency admissions	Admissions reduced (adjusted aOR = 0.41, 95% CI 0.28–0.58; *P* < 0.001); longer LOS (183 vs. 125 min; *P* < 0.001)
Holder [34] (USA)	Children and adolescents with a psychiatric disorder, aged 5–18 years	Before–after retrospective cohort	2007–2013	Staff restructuring (8 mental health social workers, training, 8-h daily psychiatrist, and psychiatric unit referrals)	Total *N* = 3220 (Before *N* = 1237, after *N* = 1983)	Informal mental health assessment by nurse or social worker	LOS in emergency dept. and emergency admissions	LOS reduced 14.7–12.1 h (*P* < 0.001). Admissions decreased (17–1%, *P* < 0.001)
Ishikawa [27] (Canada)	Children and adolescents with acute psychiatric concern, aged 0–17 years	Non-randomised control trial	2016–2019	Staff restructuring (staff training on mental health assessment and deployment at site)	Total psych visits *N* = 6576 (intervention *N* = 3467, control *N* = 3109)	Control site at another paediatric emergency dept. with RMNs	LOS in emergency dept. and 30-day readmissions	LOS reduced 85.3 min (*P* < 0.05)
Mahajan [37] (USA)	Adolescents with any psychiatric disorder, aged 9–16 years	Before–after retrospective cohort	2002	Clinical guidance training (triaged by social worker and child psychiatrist)	Total psych visits *N* = 1031 (before *N* = 568, after *N* = 412)	Assessment by emergency physician	LOS in emergency dept	LOS reduced (259.49 min ± 171.12 vs. 216.39 ± 152.95 min, *P* < 0.01)
Parker [38] (Canada)	Adolescents with a psychiatric disorder, aged < 19 years	Before–after retrospective cohort	1998–1999	Psychiatric triage and crisis support team (emergency assessment and urgent care referrals)	Total *N* = 682 (before *N* = 356, after *N* = 326)	Assessment by emergency physician and inpatient ward	Emergency admissions	Admissions reduced 6.3–2.3% (F(3,42) = 4.6, *P* < 0.05)
Reliford [39] (USA)	Children and adolescents with acute psychiatric concern, aged 3–18 years	Before–after retrospective cohort	July–Dec 2017	Telepsychiatry consultations (optional alternative to face-to-face)	Total *N* = 35	Face-to-face consultations	3-monthly mean LOS in emergency dept	LOS reduced for non-hospitalised patients (285 vs 193 h; *P* = 0.032), no reduction for hospitalised patients

*LOS* length of stay, *CI* confidence interval, *RMN* registered mental health nurse

Next, innovations embedding psychological therapies within the emergency department (see Table 2), included psychoeducation (*N* = 3), a newly established crisis unit (*N* = 3), and telephone follow-up (*N* = 2). Suicide prevention psychoeducation was delivered to caregivers of adolescents with suicidal ideation in a mixed gender and white/non-white cohort; whilst the innovation did not significantly reduce return emergency visits, there was a significant reduction in emergency admissions (32.8% pre-intervention to 24.5% post-intervention) (OR = 2.78, 95% CI 0.55–14.10, *N* = 185, *P* < 0.01) [29]. This reduction did not differ by ethnicity, age, gender, insurance type, or clinical complexity. When targeting all MHR emergencies, a psychiatric liaison program delivered by a social worker and a child psychiatrist similarly reduced emergency admissions (adjusted OR = 0.35, 95% CI 0.17–0.71, *P* < 0.01), and LOS 27% (95% CI 0–46%, *P* = 0.05) [30]. A 24-h triage and psychoeducation service reduced LOS from 332 to 244 min (*P* < 0.01) [41], but reported no change in admissions.

**Table 2:** Characteristics of psychological therapy innovations within emergency departments

Study (Country)	Population/ inclusion criteria	Study design	Dates	Intervention and resources	Participants	Comparator	Outcomes	Intervention effects
Cummings [43] (USA)	Children and adolescents with autism spectrum disorder, aged 5–24 years	Before–after retrospective cohort	2015–2016	Telephone follow-up (short-term intensive, multidisciplinary support, delivered by psychiatrist director, clinical manager and community worker)	Total *N* = 41 (emergency dept. returners *N* = 13)	Admission to acute inpatient paediatric ward (same pre- and post-cohort)	Average LOS in emergency dept.	LOS reduced 6% (315.6 vs. 298.3 h)
Greenfield [28] (Canada)	Caregivers of adolescents with a psychiatric concern, non-hospitalised (75% suicide related), aged 8–15 years	Before–after retrospective cohort	2000–2003	Telephone follow-up delivered by child psychiatrist and clinical nurse specialist (a short-term, intensive, multidisciplinary support and triage)	Total *N* = 980 (before *N* = 412, after *N* = 568)	Admission to general inpatient paediatric, medical, or surgical ward	Emergency admissions	Admissions reduced 16% (152, 37–118, 21%, OR = 0.45, 95% CI 0.33–0.60, *P* < 0.001)
Hasken [33] (USA)	Children and adolescents with a psychiatric concern, aged 2–24 years	Before–after retrospective cohort	2016–2017	Psychiatric crisis unit in the emergency dept. staffed by psych team (10-bed inpatient ward with psychological therapies)	Total *N* = 317 (before *N* = 91, after *N* = 226)	Admission to inpatient medical ward or transferred to a psychiatric unit	LOS in emergency dept. and emergency admissions	Admissions reduced 22.2–8.5% (*P* = 0.008). LOS increased 363–418 min
Parast [29] (USA)	Caregivers of children and adolescents with suicide risk, aged 5–17 years	Retrospective observational cohort	2013–2014	Psychoeducation (risk prevention delivered in emergency dept. and inpatient setting by emergency care team)	Total = 378 (emergency dept. *N* = 194; inpatient *N* = 184)	Paediatric emergency care without risk-prevention counselling	Emergency admissions	Admissions reduced (32.8–24.5%) (OR = 2.78, 95% CI 0.55–14.10, *P* < 0.01)
Sheridan [30] (USA)	Children and adolescents with a psychiatric disorder, aged < 18 years	Before–after retrospective cohort	2012–2014	Multidisciplinary triage and psychoeducation (introduction of child psychiatrist and mental health social worker)	Total *N* = 212 (before *N* = 83, after *N* = 129)	Emergency dept. triage by paediatrician or emergency care social worker	LOS in emergency dept. and emergency admissions	LOS reduced 27% (95% CI 0–46%, *P* = 0.05); admissions reduced (aOR = 0.35, 95% CI 0.17–0.71, *P* < 0.01)
Stricker [46] (USA)	Adolescents with acute psychiatric concern, age not reported	Before–after retrospective cohort	2012–2017	Complex intervention. (psych triage scale, high-acuity and low-acuity waiting rooms, urgent care unit, restraint training and MDT of psychiatrists, social workers, and psych nurses)	Post-intervention cohort visiting the emergency dept. (no raw data reported)	Two consultant paediatricians; waiting room managed by emergency care nurses	LOS in emergency dept. and emergency admissions	LOS reduced 45%, admissions reduced 20% with a multidisciplinary team and a further 20% with the urgent care centre
Rogers [40] (USA)	Children and adolescents with any psychiatric disorder, aged 5–17 years old	Before–after retrospective cohort	2006–2008	Psychiatric crisis unit (6-bed unit, MDT assessment, intensive care and stabilisation, psychiatric nursing team)	Total *N* = 3053 (Before *N* = 1190, after *N* = 1273)	On-call triage by social worker with inpatient admission or discharge	Annual mean LOS in emergency dept	LOS reduced 47.3%, (10.8 vs. 19.7 h, *P* < 0.0001)
Uspal [41] (USA)	Adolescents with a psychiatric disorder or concern, except substance-related, aged < 18 years	Before–after retrospective cohort	2010–2012	Dedicated psychiatric triage and treat team incl. psych nurse or social worker, and a practitioner (24/7, individual and family psychoeducation, discharge planning)	Total *N* = 1640 (Before *N* = 738, after *N* = 902)	Triaged by paediatrician and social workers	Annual mean LOS in emergency dept and emergency admissions	LOS reduced from 332 to 244 min (*P* < 0.001). No change in admissions

The most effective innovation in the emergency department was introducing a psychiatric crisis unit, which significantly reduced admissions between 15 and 20% [33, 46] and LOS around 45% [40, 46]. A large tertiary care hospital established an inpatient psychiatric urgent care unit adjacent to the emergency department, which reduced the rate of inpatient admissions to the paediatric acute ward from 22.2 to 8.5%, 12 months pre- and post-intervention (*P* = 0.008) [33]. This intervention increased the capacity for psychiatric assessments (226 vs 91, *P* < 0.01), yet the LOS increased by 55 min to deliver psychological therapies. There was a 40% reduction in admissions and 45% reduction in LOS after a complex intervention encompassing a psychiatric crisis unit, multidisciplinary and structured triage, and separate waiting rooms for high-acuity and low-acuity patients, though no raw data were reported. [46].

Introducing a multidisciplinary telephone follow-up service from the emergency department for patients and caregivers of MHR emergencies, consisting mainly of suicide-related emergencies, reduced admissions by 16% (OR = 0.45, 95% CI 0.33–0.60, *P* < 0.001) [28]. For LOS, another telephone follow-up study reported a monthly total LOS reduction from 315 to 298 h [43]. All psychological innovations required psychiatric staff for assessment and treatment.

Innovations embedding psychological therapies within acute inpatient settings included therapeutic skills training (*N* = 1), meal supervision (*N* = 1), and outpatient aftercare or telephone follow-up (*N* = 3), which reduced readmissions between 7.7 and 37% and inpatient LOS between 3 and 31 days (see Table 3). Inpatient innovations included a therapeutic skills group for anxiety disorders, encompassing grounding exercises, meditation, yoga, and relaxation strategies [44], which reduced 30-day readmissions (9.5%) and 90-day readmissions (15.6%) compared to individual and family psychoeducation; 74% (*N* = 64) were female and no statistical comparison was reported. A standardised meal supervision program for eating disorders (*N* = 56), compared to no supervision in the control group (*N* = 52), comprised a trained staff member at the bedside discussing patient-specific preferences, averting conversations about weight, body image, and calories [36]. Playing music or watching TV acted as distractive coping strategies. The innovation reduced LOS by 3 days (*P* = 0.27) reaching weight restoration in shorter time. Participants were predominantly from White ethnicities (89–100%) and female (82–87%).

**Table 3:** Characteristics of innovations provided within, or alongside, acute inpatient paediatric settings

Study (Country)	Population/inclusion criteria	Study design	Dates	Intervention and resources	Participants	Comparator	Outcomes	Intervention effects
McDowell [44] (USA)	Adolescents with an anxiety disorder, aged 9–17 years	Before–after retrospective cohort	2018–2019	Psychoeducation (6 × 1 h/week, incl. mindfulness) delivered by 2 psych clinicians and a yoga teacher)	Eight therapy courses, total invited *N* = 87 (non-attenders *N* = 6, 7%)	Routine individual and family therapy	30-day and 90-day emergency readmission	30-day (9.5%) and 90-day readmissions (15.6%) reduced
Huryk [35] (USA)	Adolescents with an eating disorder, aged 8–21 years	Before–after retrospective cohort	2011–2017	Psychological therapy (integration of FBT strategies and art therapy in usual 40 × 1 h PHP, delivered by family therapists and nutritionists)	Total *N* = 326 (usual PHP *N* = 188, FBT-based PHP *N* = 138)	Usual PHP including relapse prevention, and psychoeducation	3-year emergency readmission	Readmissions reduced (22, 12% vs. 4, 3%, *X*^2^(1,326) = 8.40, *P* = 0.004)
Kells [36] (USA)	Adolescents with an eating disorder, aged < 20 years	Before–after retrospective cohort	Early—late 2011	Psychological support (3 × 30-min meal supervision per day/pp)	Total *N* = 108 Intervention *N* = 56, control *N* = 52	No meal supervision	Inpatient LOS	LOS was 3 days shorter (*P* = 0.27)
Gusella [32] (Canada)	Adolescents with anorexia nervosa, aged 9–15 years	Before–after retrospective cohort	1997–2011	Outpatient FBT delivered by an RMN, a social worker, a dietician, a psychiatrist, and a psychologist	Total *N* = 46 (Before *N* = 14, after *N* = 32)	Psychoeducation family sessions	One-year emergency readmission and inpatient LOS	Reduced readmissions (34.4% vs. 71.4%, *P* = 0.03) and LOS (50 vs 19 days, *P* = 0.03)
Wallis [42] (Australia)	Adolescents with anorexia nervosa	Non-randomised controlled trial	2006–2007	40 × 1 h outpatient FBT delivered by social workers, psychologists, and a psychiatrist	Total *N* = 69 (intervention *N* = 39, control *N* = 30)	20 sessions of outpatient individual cognitive-based therapy	One-year emergency readmission	Readmissions increased (28.2% vs. 14.3%, *P* < 0.001)
Ramsbottom [45] (USA)	Caregivers of children with a psychiatric disorder, aged 2–12 years	Before–after retrospective cohort	2014–2017	Telephone follow-up delivered by an RMN manager working 36 h/week (risk-targeted case management)	Total *N* reviewed = 1316 (contacted N = 1221, 92.7%)	Contacted without case management	30-day emergency readmission	Readmissions reduced (year 1 = 29.5%, Year 2 = 7.9%, Year 3 = 5%, *P* = 0.03)
Carlisle [25] (Canada)	Adolescents with a psychiatric disorder or self-inflicted harm, aged 15–19 years	Retrospective observation-al cohort	2002–2004	Aftercare with primary care physician or psychiatrist as referral from inpatient setting (30-day follow-up or outpatient clinic)	Total *N* = 3004 (aftercare *N* = 1502, no aftercare *N* = 1502)	Discharged without aftercare	One-year emergency readmission	Readmissions increased (283, 19% vs. 222, 15%, aHR = 1.38, 95% CI 1.14–1.66, *P* < 0.001)
Cheng [31] (Canada)	Children and adolescents with a psychiatric disorder, aged 5–17 years	Retrospective observational cohort	2007–2012	Aftercare with psych outpatient clinic as referral from inpatient setting (90-day follow-up)	Total *N* = 15,628 (Aftercare *N* = 10,461, no aftercare *N* = 5167)	Discharged without aftercare	90-day emergency readmission	Readmissions reduced 32% (aHR = 0.68, 95% CI 0.58–0.80, *P* < 0.001)

*FBT* family-based therapy, *PHP* partial hospitalisation program, *X*^2^ Chi-squared, *LOS* length of stay, *aHR* adjusted hazard ratio, *CI* confidence interval, *RMN* registered mental health nurse

For integrated pathways, introducing family-based therapy (FBT) to an existing partial hospitalisation program (PHP) for eating disorders reduced 3-year readmissions by 7.7% [35]. The FBT innovation used family-based psychotherapy and art therapy (*N* = 138), compared to traditional PHP, involving relapse prevention, goal setting, and neurobiology psychoeducation, which significantly reduced readmission (*X*^*2*^ (1,326) = 8.40, *P* = 0.004, *N* = 188). Two further studies evaluated outpatient FBT, specifically for individuals with anorexia nervosa. Family-based psychotherapy compared to a control group of psychoeducational family sessions [32] significantly reduced readmissions for weight restoration (34.4 vs. 71.4%, *P* = 0.03). In the second study, both intervention (*N* = 32) and control (*N* = 16) groups received 20 FBT sessions, yet the intervention group received an additional 20 FBT psychotherapy sessions, whereas controls received individual cognitive-based therapy (CBT) [42]. The prolonged FBT group had significantly higher 12-month readmissions (28.2 vs. 14.3%, *P* < 0.001), albeit shorter readmission LOS (mean days = 71.93, SD = 68.28) than the controls (mean days = 12.51, SD = 24.67, *P* < 0.001). Thus, FBT treatment modality and length of therapy mattered. In both studies, participants were predominantly female (94–95%), and commonly had comorbidity (21.7% mood disorders, 19.6% anxiety disorders) [42] and eating disorder psychopathology. [32]

Two Canadian studies compared those who received outpatient aftercare comprising any follow-up assessment, onward referral, residential treatment, or physician service, compared to those who received no aftercare, and reported contrasting findings. One recorded a 32% reduction in 90-day readmissions (adjusted hazard ratio, aHR = 0.68, 95% CI 0.58–0.80, *P* < 0.001, *N* = 15,628) [31]; the other a 38% increase in risk of 1-year readmission (aHR = 1.38, 95% CI 1.14–1.66, *P* < 0.001, *N* = 3004) [25]. However, these contrasting findings might explain more about short- and long-term readmission in this cohort rather than the intervention effects of general outpatient follow-up, given that the 'no aftercare' groups were allocated based on lower clinical acuity, thus, causing allocation bias. Alternatively, an integrated intervention for ‘troubleshoot’ telephone follow-up service was provided for caregivers (*N* = 1211) [45]. The modal recipient groups were those with depressive, attention deficit hyperactivity (ADHD), anxiety, post-traumatic stress (PTSD), and autism spectrum disorders due to high risk of readmission. This reduced 30-day readmission by 29.5% in year 1, 7.9% in year 2, and 5.1% in year 3.

This review summarises evidence for healthcare innovations within three paediatric emergency settings. First, within emergency departments: multidisciplinary and telepsychiatry triage both reduced LOS in the emergency department and emergency admissions; however, telepsychiatry showed propensity to increase time spent in the emergency department. Early intervention therapy in the emergency department, either through short-term psychoeducation, telephone follow-up, or a dedicated psychiatric unit for high-acuity patients reduced admissions between 10 and 35%, which simultaneously reduced waiting times. Second, on the acute ward, psychological therapy for anxiety disorders and guided meal supervision for eating disorders showed promise for reducing LOS and readmissions; yet robust methods were lacking. Third, integrated interventions alongside hospitalisation signposted adolescents with eating disorders to FBT delivered as partial hospitalisation, which reduced readmissions by 9–37%. However, prolonging FBT up to 40 sessions compared to 20 sessions increased readmissions by 14%. This evidence suggests that engaging caregivers of adolescents with MHR crises in psychological intervention helped adolescents to avoid institutionalisation within medical hospitals.

## Discussion

This is the first evidence synthesis for healthcare innovations of integrated care for young people with MHR emergencies to reduce emergency hospitalisation and rehospitalisation, and length of hospital stay—that is, to get well quickly and stay well enough to remain out of hospital. These models of integrated care focussed on embedding psychiatric consultations to improve triage into acute hospitalisation and embedding psychological therapies into typically medical services to improve integrated pathways within inpatient services. Innovations for integrated care and integrated pathways set out to improve the comprehensiveness of care for medical emergencies with aetiology in mental disorder [3], such as self-inflicted harm, eating disorders, and intoxication. Innovations for integrated care show promising evidence for reducing the rate of emergency admissions to an acute ward, the LOS in both the emergency department and inpatient setting, and the rehospitalisation rate after discharge. Thus, integrated care not only improves health outcomes [17, 18] and effectively reduces psychiatric hospitalisation [19]—this review adds that signposting to psychological interventions also reduces emergency readmissions in paediatric emergency settings too. Whilst there is sometimes no alternative to hospitalisation for medical emergencies [16, 17], integrating psychiatric care into emergency services helps to triage effectively, intervene earlier, and signpost to therapeutic support, reducing lengthy and repeat hospitalisation.

Engaging family members or guardians was an innovative treatment modality for specific MHR emergencies. This was evident in innovations providing FBT aftercare for eating disorders [32, 35], risk-prevention psychoeducation for guardians of individuals with suicide-related emergencies [29], and risk-targeted follow-up for caregivers of individuals with suicidality, PTSD, ADHD, or autism [28, 45]. The important role of parental figures in aftercare therapies and recovery might be associated with their related risk factors, which are commonly deprivations such as parental mental illness, childhood trauma (ACEs), conflict in the home, and socioeconomic deprivation [49].

During the COVID-19 pandemic, there were more medical emergencies for eating disorders, but fewer for suicidality and substance-related disorders [5], which might be due to increased time spent at home and the absence of school and community activities, respectively. Designing and testing psychological interventions for integrated care should consider differing response styles between clinical cohorts, as well as adjust for annual fluctuations in emergency service utilisation during the COVID-19 pandemic, using multilevel or stratified intervention effects.

Our review identified evidence gaps for what innovations are effective for whom, and in what settings. For instance, family-based therapy delivered as aftercare from acute settings (i.e. partial hospitalisation) for adolescents with eating disorders consistently reduced ‘the revolving door’ of repeat admissions. Yet, robust evidence of psychoeducation for acute inpatients with anxiety-related and eating disorders was limited. Few studies reported ethnicity characteristics of the sample, nor tested intervention effects for individual differences such as ethnicity and gender. Most studies were in predominantly White female populations; thus, socio-demographic differences in responses to treatment modalities are not yet known. This further evidenced the White centricity within mental health research, particularly in paediatrics [50]. Given that all studies involved naturalistic hospital data, this lack of nuance may be due to ethnic disparities in access to psychiatric services [51].

Methodologically, before–after intervention comparisons of hospital data were the typical study design, which sought to mimic a randomised control trial given the homogeneity of setting and participants in the intervention and control groups. However, annual changes in service use [5] demonstrates the need to adjust for individual differences between the pre- and post-intervention cohorts, which was often missing in studies reviewed. Propensity score matching is a robust approach to adjust for these variations, being careful of allocation bias. Better still, future designs should report intervention effects by gender, ethnicity, and clinical cohorts, to evidence what works for whom, and to what effect.

To encourage commissioning, future evaluations must report the resources required for establishing integrated care and pathways, to permit accurate cost–benefit analyses for reducing hospitalisation. All studies we reviewed required multidisciplinary staffing at minimum, and new capital facilities at most. The co-location of psychiatry space as well as expertise, alongside acuity risk triage were key components of reducing over-medicalisation of MHR emergencies and improving access to psychotherapies. Most studies we reviewed were conducted in the USA or Canada. This might be due to country characteristics using private and social insurance as dominant health expenditures [52]. Competitive markets incentivise care providers to be cost-effective. As the UK further implements decentralised commissioning [12], we might see more evaluative research of this kind. However, comprehensive and integrated care is not synonymous with competitive markets [53], and it will be important to keep patient safety, care integration, and quality of care as the rationale for improving efficiency—synonymous with increasing service capacity [13].

## Supplementary Information

Below is the link to the electronic supplementary material.

Supplementary file1 (DOCX 32 KB)

Supplementary file2 (DOCX 29 KB)

## Data Availability

MO had full access to all the data in the study and takes responsibility for the integrity of the data and the accuracy of the data analysis. All data collected for this article, including data extraction tables, are included in this published article in the tables and supplementary files.
