# Supplementary material for: Models of integrated care for young people experiencing medical emergencies related to mental illness: a realist systematic review
Source: Eur Child Adolesc Psychiatry. 2022 Sep 24;32(12):2439–52. doi: 10.1007/s00787-022-02085-5 (PMC9510153; doi:10.1007/s00787-022-02085-5)
Supplement: Supplementary file 1 — Supplementary file1 (DOCX 32 KB) [file 787_2022_2085_MOESM1_ESM.docx]

Supplementary table 1. Search strategy for all databases (Otis et al., 2021)

| Concept | Database | | | | | | |
| --- | --- | --- | --- | --- | --- | --- | --- |
|  | Embase Classic + Embase  <1990 to Jun 10, 2021> | | Ovid Psychinfo  <1990 to June 10, 2021> | | Ovid MEDLINE(R) ALL <1990 to May 28, 2021> | | Web of Science  <1990-June 15, 2021> |
|  | Free text terms *(number of returns* | Mesh terms  *(number of returns)* | Free text terms *(number of returns* | Mesh terms  *(number of returns)* | Free text terms *(number of returns* | Mesh terms  *(number of returns)* | Free text terms  *(number of returns)* |
| Young people aged 8-18 years | child*.mp. (3144377)  adolesc*.mp. (1843089)  youth*.mp. (106403)  p?ediatric*.mp. (700736)  teen*.mp. (45533) | adolescent/ or child/ (3037421)  pediatrics/ or pediatric emergency medicine/ (91516)  young people.mp. (43109)  exp Child Psychiatry/ exp Child Psychology/ (42358)  exp Adolescent Psychiatry/ or exp Adolescent Psychotherapy/ or exp Adolescent Psychology/ (42358)  pediatrics/ or chronically ill children/ (90654) | child*.mp.(3144377)  adolesc*.mp.(1843089)  youth*.mp.(106403)  p?ediatric*.mp.(700736)  teen*.mp.(45533) | adolescent/ or child/ (3037421)  pediatrics/ or pediatric emergency medicine/ (91516)  young people.mp.(43109)  exp Child Psychiatry/ or exp Child Psychotherapy/ or exp Child Psychology/ (42358)  exp Adolescent Psychiatry/ or exp Adolescent Psychotherapy/ or exp Adolescent Psychology/ (42358)  pediatrics/ or chronically ill children/ (90654) | child*.mp. (2531737)  adolesc*.mp. (2176313)  youth*.mp. (87932)  p?ediatric*.mp. (435781)  teen*.mp. (32205)  young people.mp. (29797) | adolescent/ or child/ (2904302)  pediatrics/or pediatric emergency medicine/ (55835) | child* (1,242,308)  adolesc* (343,684)  youth* (124,731)  paediatric* (45,942)  teen* (27,962)  young people (74,624)  “Child Psychology” (402)  “Child Psychotherapy” (293)  “Adolescent Psychiatry” (1,328)  “Adolescent Psychotherapy” (191)  “Adolescent Psychology” (138)  “chronically ill child*” (423) |
| Mental Illness | (mental adj3 problem*).mp.  (23205)  (mental adj3 cris?s).mp. (1090)  psychiatr*,mp. (462492)  mental illness*.mp. (47146)  somat*.mp. (437997)  malinger*.mp. (4429)  autis*.mp. (84132)  eating disorder*.mp. (41081)  rumination disorder*.mp. (48)  abnormal behavio?r*.mp. (5741)  unusual behavio?r*.mp. (1228)  “substance use disorder”/ (49032)  unexplained behavio?r*.mp. (40)  unexplained symptom*.mp. (2010)  self?harm.mp.(235)  suicid*.mp. (145570)  substance ?use.mp. (55149)  alcohol*.mp. (744792)  cannab*.mp. (86078)  overdose*.mp. (48703)  mental disorder*.mp. (81124) | anorexia.mp. (97588)  bulimia.mp. (16448)  purging.mp. (6256)  restrictive food intake.mp. (416)  pica.mp. (4227)  drug abuse.mp. (80904)  psychosomatic.mp. (32717)  Substance-Related Disorders/ (30117)  mental disorders/ or neurotic disorders/ or obsessive-compulsive disorder/ or panic disorder/ or "bipolar and related disorders"/ or "disruptive, impulse control, and conduct disorders"/ or dissociative disorders/ or "feeding and eating disorders"/ or amnesia/ or cognition disorders/ or "attention deficit and disruptive behavior disorders"/ or child behavior disorders/ or child development disorders, pervasive/ or schizophrenia, childhood/ or personality disorders/ or "schizophrenia spectrum and other psychotic disorders"/ or somatoform disorders/ or substance-related disorders/ or "trauma and stressor related disorders"/ (638604)  exp Mental Disorders/ (2462294)  mental disease/ (259234)  mental disease.mp. or exp mental disease/ (2463167)  attempted suicide/ (34873)  exp Eating Disorders/ (56991)  exp Somatoform Pain Disorder/ or exp Somatoform Disorders/ (28984) | (mental adj3 problem*).mp.(23205)  (mental adj3 cris?s).mp. (1090)  psychiatr*.mp.(462492)  mental illness*.mp.(47146)  somat*.mp.(437997)  malinger*.mp.(4429)  autis*.mp.(84132)  eating disorder*.mp.(41081)  rumination disorder*.mp.(48)  abnormal behavio?r*.mp.(5741)  unusual behavio?r*.mp.(1228)  unexplained behavio?r*.mp.(40)  unexplained symptom*.mp.(2010)  self?harm.mp.(235)  suicid*.mp.(145570)  substance ?use.mp.(55149)  alcohol*.mp.(744792)  cannab*.mp.(86078)  overdose*.mp.(48703)  mental disorder*.mp.(81124)  "substance use disorder"/ (49032) | mental disease.mp. or exp mental disease/ (2463167)  psychosomatic.mp. (32717)  anorexia.mp.(97588)  bulimia.mp.(16448)  purging.mp.(6256)  restrictive food intake.mp.(416)  pica.mp.(4227)  drug abuse.mp. (80904)  Substance-Related Disorders/ (30117)  mental disorders/ or neurotic disorders/ or obsessive-compulsive disorder/ or panic disorder/ or "bipolar and related disorders"/ or "disruptive, impulse control, and conduct disorders"/ or dissociative disorders/ or "feeding and eating disorders"/ or amnesia/ or cognition disorders/ or "attention deficit and disruptive behavior disorders"/ or child behavior disorders/ or child development disorders, pervasive/ or schizophrenia, childhood/ or personality disorders/ or "schizophrenia spectrum and other psychotic disorders"/ or somatoform disorders/ or substance-related disorders/ or "trauma and stressor related disorders"/ (638604)  exp Mental Disorders/ (2462294)  attempted suicide/ (34873)  exp Eating Disorders/ (56991)  exp Somatoform Pain Disorder/ or exp Somatoform Disorders/ (28984)  mental disease/ (259234) | (mental adj3 problem*).mp. (18383)  (mental adj3 cris?s).mp. (885)  psychiatr*.mp. (368392)  mental illness*.mp.(34306)  somat*.mp. (245282)  psychosomatic.mp. (16280)  malinger*.mp. (3605)  autis*.mp. (56831)  binge?eating.mp. (3)  rumination disorder*.mp. (33)  abnormal behavio?r*.mp. (3316)  unusual behavio?r*.mp. (1175)  unexplained behavio?r*.mp. (29)  unexplained symptom*.mp. (1621)  self?harm.mp. (32)  suicid*.mp. (100591)  substance ?use.mp. (40745)  alcohol*.mp. (442788)  cannab*.mp. (45704)  overdose*.mp. (26536) | anorexia.mp. (35702)  bulimia.mp. (11049)  purging.mp. (5257)  restrictive food intake.mp. (270)  pica.mp. (3210)  drug abuse.mp (18255)  Substance-Related Disorders/ (97957)  mental disorders/ or “bipolar and related disorders” /or “disruptive, impulse control, and conduct disorders”/ or dissociative disorders/ or “feeding and eating disorders”/ or amnesia/ or cognition disorders/ or “attention deficit and disruptive behaviour disorders”/ or child behaviour disorders/ or child development disorders, pervasive/ or schizophrenia, childhood/ or neurotic disorders/ or somatoform disorders/ or substance-related disorders/ or “trauma and stressor related disorders” /or “behavioural disciplines and activities”/ (394159) | mental near/2 problem* (17,296)  mental near/2 cris* (631)  psychiatr* (166,775)  mental illness* (52,265)  somat* (187,023)  “psychosomatic” (4,206)  malinger* (1,826)  autis* (54, 326)  anorexia (26, 139)  purging (12, 343)  “eating disorder” (11,182)  “restrictive food intake” (187)  “rumination disorder” (22)  pica (3,572)  “abnormal behaviour*” (960)  “unusual behaviour*” (846)  “abnormal behavior*” (3,020)  “unusual behavior*” (2,605)  “unexplained behaviour*” (15)  “unexplained behavior*” (34)  “unexplained symptom*” (1,402)  “self harm” (6,850)  self-harm (7,158)  suicid* (67,148)  “substance use*” (55,822)  “substance abuse” (29,277)  alcohol* (444,926)  cannab* (34,865)  overdose* (16,515)  “drug abuse” (16,009)  “substance-related disorders” (1,218)  “neurotic disorder*” (398)  “mental disorder*” (39,098)  “obsessive-compulsive disorder” (14,809)  “panic disorder” (11,592)  “bipolar and related disorders” (27)  “disruptive, impulse control and conduct disorders” (8)  “feeding and eating disorders” (212)  amnesia (10,888)  “cognition disorders” (423)  “attention deficit and disruptive behavior disorders” (26)  “child behavior disorders” (45)  “attention deficit and disruptive behaviour disorders” (9)  “dissociative disorder*” (782)  “child behaviour disorders” (11)  “child development disorders, pervasive” (29)  schizophrenia, childhood (4,480)  personality disorders (39,783)  “schizophrenia spectrum and other psychotic disorders” (54)  “somatoform disorder*” (1,929)  “substance-related disorders” (1,218)  “trauma and stressor related disorders” (63)  bulimi* (10,889)  “binge eating” (5,590)  “behavioural disciplines and activities” (9)  “attempted suicide” (3,908)  “somatoform pain disorder” (131) |
| Acute care settings | emergency admission*.mp. (4189)  emergency readmission*.mp.(288)  rehospitali*.mp. (11246)  (hospital adj3 emergenc*).mp. (26661)  (emergenc* adj2 medic*).mp.(86187)  acute hospital*.mp.(9515)  (acute adj2 medic*).mp.(10497)  (emergenc* adj2 treatment*).mp.(26214)  re?feeding.mp.(7368)  (emergenc* adj2 in?patient).mp. (2258)  (non?elective adj2 care).mp. (12)  (non?elective adj2 treatment).mp. (68)  (unscheduled adj2 care).mp. (429)  (unscheduled adj2 treatment).mp. (64)  (unscheduled adj2 medical).mp. (123)  (non?elective adj2 medical).mp. (11)  (unplanned adj2 care).mp. (603)  (unplanned adj2 treatment).mp. (228)  (unplanned adj2 medical).mp. (141)  (prospective adj2 care).mp. (1211)  (prospective adj2 treatment).mp. (3069)  (prospective adj2 medical).mp. (429)  (urgent adj2 care).mp. (4866)  (urgent adj2 treatment).mp. (2659)  (urgent adj2 medical).mp.(1419)  emergency hospitali?ation*.mp. (651)  acute hospitali?ation.mp. (1444)  acute treatment*.mp. (13333)  acute admission*.mp. (1709) | Emergency Service, Hospital/ (5899)  emergency medicine/ or pediatric emergency medicine/ (43461)  emergency medicine/ (42600)  emergency treatment/ (17951)  acute care.mp. (34095)  acute ward.mp. (395)  emergency services/ or crisis intervention services/ or emergency medicine/ (42603)  hospital emergency service/ or emergency health service/ (112581) | emergency admission*.mp. (4189)  emergency readmission*.mp. (288)  rehospitali*.mp.(11246)  (hospital adj3 emergenc*).mp.(26661)  (emergenc* adj2 medic*).mp.(86187)  acute hospital*.mp.(9515)  (acute adj2 medic*).mp.(10497)  (emergenc* adj2 treatment*).mp.(26214)  re?feeding.mp.(7368)  (emergenc* adj2 in?patient).mp.(2258)  (non?elective adj2 care).mp.(12)  (non?elective adj2 treatment).mp.(68)  (unscheduled adj2 care).mp.(429)  (unscheduled adj2 treatment).mp.(64)  (unscheduled adj2 medical).mp.(123)  (non?elective adj2 medical).mp.(11)  (unplanned adj2 care).mp.(603)  (unplanned adj2 treatment).mp.(228)  (unplanned adj2 medical).mp. (141)  (prospective adj2 care).mp.(1211)  (prospective adj2 treatment).mp.(3069)  (prospective adj2 medical).mp.(429)  (urgent adj2 care).mp.(4866)  (urgent adj2 treatment).mp.(2659)  (urgent adj2 medical).mp.(1419)  emergency hospitali?ation*.mp.(651)  acute hospitali?ation.mp.(1444)  acute treatment*.mp.(13340)  acute admission*.mp.(1709) | Emergency Service, Hospital/ (5899)  emergency medicine/ or pediatric emergency medicine/ (43461)  acute care.mp. (34095)  acute ward.mp.(395)  emergency services/ or crisis intervention services/or emergency medicine/ (42603)  hospital emergency service/ or emergency health service/ (112581)  emergency medicine/ (42600)  emergency treatment/ (17951) | (length of stay adj3 acute).mp. (525)  emergency admission*.mp. (2501)  emergency readmission*.mp. (187)  rehospitali*.mp. (6747)  (hospital* adj4 emergenc*).mp. (91801)  (emergenc* adj2 medic*).mp. (85659)  acute hospital*.mp. (5955)  (acute adj2 medic*).mp. (6536)  (emergenc* adj2 treatment*).mp. (17791)  medical stabili?ation.mp (235)  re?feeding.mp. (5216)  (acute adj2 in?patient*).mp. (2759)  (emergenc* adj2 in?patient*).mp. (1280)  (hospital adj2 readmi*).mp. (8740)  (non?elective adj2 care).mp. (9)  (non?elective adj2 admission*).mp. (120)  (non?elective adj2 treatment).mp. (50)  (non?elective adj2 readmission*).mp. (54)  (unscheduled adj2 care).mp. (276)  (unscheduled adj2 admission*).mp. (191)  (unscheduled adj2 readmission*).mp. (84)  (unscheduled adj2 treatment).mp. (43)  (unscheduled adj2 hospital*).mp. (254)  (unplanned adj2 hospital*).mp. (1391)  (unplanned adj2 admi*).mp. (1207)  (unplanned adj2 care).mp. (399)  (unplanned adj2 treatment).mp. (118)  (unplanned adj2 readmi*).mp. (1754)  (non?elective adj2 hospital*).mp.  (84)  (prospective adj2 care).mp. (810)  (prospective adj2 admission*).mp. (112)  (prospective adj2 treatment).mp. (1897)  (prospective adj2 readmission*).mp. (33)  (urgent adj2 care).mp. (3084)  (urgent adj2 admission*).mp. (366)  (urgent adj2 readmission*).mp. (49)  (urgent adj2 treatment).mp. (1651) | hospitalisation/ or “length of stay” / or patient admission/ or patient readmission (2229835)  emergency medicine/ or pediatric emergency medicine/ (14314)  acute care.mp. (23866)  Emergency Service, Hospital (74191)  Child, Hospitalized/ or Adolescent, Hospitalized/(7180) | “acute care” (17,282)  “emergency admission*” (1,732)  “emergency readmission*” (162)  rehospitali* (6,115)  hospital near/2 emergenc* (10,463)  emergenc* near/1 medic* (27,073)  “acute hospital*” (4,188)  “emergency service, hospital” (235)  “emergency medicine” (12,476)  “pediatric emergency medicine” (724)  emergenc* near/1 treatment* (4,123)  “medical stabil?ation”  (176)  refeeding (3,974)  emergenc* near/1 inpatient* (1,056)  nonelective near/1 care (7)  nonelective near/1 treatment (4)  unscheduled near/1 medical (65)  nonelective near/1 medical (4)  unplanned near/1 care (306)  unplanned near/1 treatment (99)  acute near/1 medic* (3,993)  unscheduled near/1 care (195)  unscheduled near/1 treatment (36)  unplanned near/1 medical (69)  prospective near/1 care (633)  prospective near/1 treatment (1,547)  prospective near/1 medical (204)  urgent near/1 care (2,019)  urgent near/1 treatment (958)  urgent near/1 medical (515)  hospitali* (203,085)  length of stay near/2 acute (441)  acute near/1 inpatient* (2,099)  hospital near/1 readmi* (7,892)  nonelective near/1 admission* (110)  nonelective near/1 readmission* (47)  unscheduled near/1 admission* (139)  unscheduled near/1 readmission* (68)  unscheduled near/1 hospital* (206)  unplanned near/1 hospital* (1,172)  unplanned near/1 admi* (955)  unplanned near/1 readmi* (1,602)  nonelective near/1 hospital* (73)  Child, Hospitalised (16, 341)  Adolescent, Hospitalised (2,288)  prospective near/1 admission* (94)  prospective near/1 readmission* (30)  urgent near/1 admission* (253)  urgent near/1 readmission* (41)  “emergency hospitalisation*” (51)  “emergency hospitalization*” (193)  “acute hospitalisation*” (104)  “acute hospitalization*” (704)  “acute treatment*” (6,177)  “acute admission*” (640)  “acute ward” (165)  “emergency services” (3,299)  “crisis intervention services” (31) |
| Length of stay/ readmissions | readmission*.mp. (84735)  out of area admission*.mp. (2)  out of area transfer*.mp. (1)  out of region transfer*.mp. (3)  (regional adj2 displacement).mp. (88) | “length of stay”/ or patient readmission/(255947)  “length of stay”/ (211372)  length of stay.mp. (227816)  hospital readmission/ (74205) | Readmission*.mp.(84735)  out of area admission*.mp.(2)  out of area transfer*.mp.(1)  out of region transfer*.mp.(3)  (regional adj2 displacement).mp.(88) | “length of stay”/ or patient readmission/ (255947)  length of stay.mp.(227816)  “length of stay”/ (211372)  hospital readmission/ (74205) | (care adj2 pathway*).mp. (6463)  (care adj2 trans*).mp.(14390)  (care adj2 integrat*).mp. (23374)  discharge plan*.mp.(3953)  care link*.mp.(294)  (community adj2 link*).mp.(1233)  (integrated adj2 pathway*).mp.(1295)  community partnership*.mp.(1556)  (multi?disciplinary adj2 assess*).mp.(1155)  multi?disciplinary team*.mp. (20094)  partial hospitali?ation.mp. (435)  (digital adj2 intervention*).mp.(1209) | onward care.mp.(4)  social prescribing.mp.(199)  telemedicine.mp.(35796)  “Delivery of Health Care, Integrated”/ (13389) | “hospital readmission” (4,698)  “length of stay” (53,137)  “readmission*” (27,079)  out of region admission* (684)  out-of-region admission* (1)  out of area admission* (1,629)  out-of-area admission* (12)  out of area transfer* (8,610)  out-of-area transfer* (7)  out of region transfer* (10,072)  out-of-region transfer* (9)  regional near/1 displacement (132)  care near/1 pathway* (4,346)  care near/1 trans* (9,930)  care near/1 integrat* (9,183)  “onward care” (3)  “discharge plan*” (2,307)  “care link*” (228)  community near/1 link* (2,108)  integrated near/1 pathway* (1,688)  community partnership* (16,601)  multi-disciplinary near/1 assess* (86)  multidisciplinary near/1 assess* (863)  multi-disciplinary team* (1,982)  multidisciplinary team* (18,634)  partial hospitalisation (2,368)  social prescribing (5,015)  telemedicine (13,637)  digital near/1 intervention* (864)  “Delivery of Health Care, Integrated” (46)  rehospitali?ation* (5,791) |

1. all the subject headings and keywords meaning *acute medical treatment*Combined with OR
2. all the subject headings and keywords meaning *young people*Combined with OR
3. all the subject headings and keywords meaning *mental illness* Combined with OR
4. all the subject headings and keywords meaning *length of stay, readmissions, and out-of-region admissions c*ombined with OR
5. 1 AND 2 AND 3 AND 4

Limitations: year= “1990-Current”
