# Supplementary material for: Models of integrated care for young people experiencing medical emergencies related to mental illness: a realist systematic review
Source: Eur Child Adolesc Psychiatry. 2022 Sep 24;32(12):2439–52. doi: 10.1007/s00787-022-02085-5 (PMC9510153; doi:10.1007/s00787-022-02085-5)
Supplement: Supplementary file 2 — Supplementary file2 (DOCX 29 KB) [file 787_2022_2085_MOESM2_ESM.docx]

Supplementary table 2: Quality Assessment using the modified Newcastle-Ottawa scale of included case-control studies in systematic review of strengthening models of acute care for young people presenting with symptoms indicating mental illness

(A study can be awarded a maximum of one star (*) for each numbered item within the Selection and Outcome categories. A maximum of two stars (**) can be given for Comparability)

|  | Selection | | | Comparability | Outcome | | | |  |
| --- | --- | --- | --- | --- | --- | --- | --- | --- | --- |
| First Author, Date (Country) | Representativeness of exposed cohort (Maximum: *) | Selection of non-exposed cohort  (Maximum: *) | Ascertainment of exposure  (Maximum: *) | Comparability of cohorts  (Maximum: **) | Assessment of outcome  (Maximum: *) | Was follow-up long enough for outcomes to occur? | Adequacy of follow up of cohorts  (Maximum: *) | Total Score (out of 8) | Power |
| Stricker | A* | A* | A* | 0, no p value. | A* | A* one-five years (multi-phased) | A* | 6/8 | Poor, comparability |
| Cummings | B* | A* | A* | 0, no p value. | A* | A* one year | B* 5% omitted (2 of 41) | 6/8 | Poor, comparability |
| Holder | A* | A* | A* | B*, P value only | A* | A* 30-day, over 3 years | A* | 7/8 | Fair |
| Ishikawa | A* | A* | A* | A*, controls for volume and acuity of all ED visits, time, and site (ITS). | A* | A*, 30-day over one year | A* | 8/8 | Good |
| Mahajan | A* | A* | A* | B*, P value only | A* | A* one year | A* | 7/8 | Fair |
| Reliford | A* | A* | A* | B*, P value only | B* | A*, 3 months | A* | 7/8 | Fair |
| Rogers | A* | A* | A* | B*, P value only | A* | A*, 1 year | A* | 7/8 | Fair |
| Uspal | A* | A* | A* | B*, P value only | A* | A*, 1 year | A* | 7/8 | Fair |
| Huryk | A* | A* | A* | 1*, P value only | A* | A* | A* | 7/8 | Fair |
| Wallis | A* | A* | A* | B* P value only, Not randomised* | A* | 12 month* | B*, 14% lost to drop out | 7/8 | Fair |
| Kells | A* | A* | A* | 1*, P value only | A* | Real time LoS* | A*, None* | 7/8 | Fair |
| Ramsbottom | A* | A* | A* | 0, no confounders or matching or stats comparison | B* | A*<30 day readmission (sept 2016-july2017) | A*, None* | 6/8 | Poor, comparability & outcome time |
| McDowell | A* | A* | A* | 0, no confounders or matching or stats comparison | A* | A*, 30-60 day readmission | A*, None* | 6/8 | Poor, comparability |
| Carlisle | A* | A* | A* | ab** propensity score matching, age, gender, diagnosis, comorbidity, income index, previous service use, health locality | B* | A*,12 months | A*, None* | 8/8 | Good |
| Cheng | A* | A* | A* | A*, risk ratios and CI | A* | A*, 90 Days | A*, None | 7/8 | Fair |
| Gusella | A* | A* | A* | A*, P value, with Fisher’s exact test for between group comparison of IVs. | A* | A*,12 months | A*, None* | 7/8 | Fair |
| Parast | B*  65% non-response rate at initial recruitment. | A* | B* | Ab**, controls for age, gender, hospital, insurance, medical complexity, caregiver ethnicity | C* | A*, 68-400 days, recruited through both ED and inpatient | A*, none/na. | 8/8 | Good |
| Greenfield | A* | A* | A* | B* (P value only) | A* | A* 3-yr follow up | A*, None* | 7-8 | Fair |
| Parker | A* | A* | A* | A** (ITS) | A* | A* 2-year pre-post | A*, none | 8 | Good |
| Hasken | A* | A* | A* | B* | A* | A* 6 months | A*, None* | 7/8 | Fair |
| Sheridan | A* | A* | A* | A** Controls for age, diagnosis, clinical history etc. | A* | A* (instant admission)  2 yr pre, 4-month post. | A*, none* | 8/8 | Good |
| Desai | A* | A* (am vs pm - potential time of day effect?) | A* | A** Adjusted for age & diagnosis; controlled allocation by am/pm | A* | A* (instant admission) one-year study | A*, none* | 8/8 | Good |
| **Scoring criteria** | **a) truly representative of the average _______________ (describe) in the community**  **b) somewhat representative of the average ______________ in the community**  **c) selected group of users e.g. nurses, volunteers**  **d) no description of the derivation of the cohort** | **a) drawn from the same community as the exposed cohort**  **b) drawn from a different source**  **c) no description of the derivation of the non-exposed cohort** | **a) secure record (e.g. surgical records)**  **b) structured interview**  **c) written self-report**  **d) no description** | **a)** study controls for other factors related to readmission & LoS (diagnosis, illness severity); or uses score matching; or uses interrupted time series analysis (ITS) with at least 2 time points pre and post; or uses group randomisation, or adjusted risk with CI**  **b)* study gives P value comparison using an uncontrolled before-after intervention; or unadjusted Risk with CI** | **a) independent blind assessment**  **b) record linkage**  **c) self-report**  **d) no description** | **a) yes (select an adequate follow up period for outcome of interest)**   - **<30 days (not long enough/ poor)** - **3 months (good)** - **12 months (very good)**     **b) no** | **a) complete follow up - all subjects accounted for**  **b)subjects lost to follow up unlikely to introduce bias - small number lost - > ____ % (select an adequate %) follow up, or description provided of those lost)**  **c) follow up rate < ____% (select an adequate %) and no description of those lost**  **d) no statement** |  |  |
